# The “more is better” fallacy in pre-service teacher training: a positive psychology perspective on the resilience-building process and job satisfaction

**DOI:** 10.3389/fpsyg.2026.1885723

**Published:** 2026-06-19

**Authors:** Lu Xing, Xueqi Wu

**Affiliations:** 1 School of Preschool Education, Changsha Normal University, Changsha, China; 2 School of Education, Faculty of Social Sciences Leisure Management, Taylor's University, Subang Jaya, Malaysia; 3 Taylor’s Culinary Institute, Faculty of Social Sciences Leisure Management, Taylor’s University, Subang Jaya, Malaysia

**Keywords:** job demands-resources model, personal resources, person-organization fit, positive psychology, pre-service teachers, teachers’ job satisfaction, teaching self-efficacy

## Abstract

The transition from academic training to practical teaching is a highly demanding phase that is critical to the psychological resilience and long-term well-being of pre-service early childhood educators. While positive psychology emphasizes the interplay between individual agency and contextual support in fostering adaptation, the specific occupational factors associated with pre-service teachers’ resilience remain underexplored. Drawing upon the motivational pathway of the Job Demands-Resources (JD-R) model, this study investigates how Person-Organization (P-O) fit (a protective contextual resource) and internship length (a potential systemic risk factor) interact to relate to job satisfaction, specifically through the mediating pathway of teaching self-efficacy (acting as a crucial personal resource). A quantitative cross-sectional design was employed, collecting valid survey data from 497 pre-service early childhood teachers (males 11.07%, females 88.93%) in China. Structural Equation Modeling (SEM) was utilized to examine the direct, mediation, and moderation effects. The structural path analysis reveals that P-O fit is significantly and positively associated with pre-service teachers’ job satisfaction. Furthermore, this relationship is partially mediated by teaching self-efficacy, which serves as a vital personal resource for resilient adaptation. Guided by Conservation of Resources (COR) theory, the study indicates that while P-O fit provides a linear buffering effect against temporal demands, the direct effects of internship length follow a prominent inverted U-shaped non-linear trajectory. The optimal threshold in our sample is estimated at 11.03 weeks, beyond which excessive practicum duration is directly linked to diminished teaching self-efficacy regardless of organizational alignment. By challenging the “more is better” assumption regarding internship duration, this study contributes valuable empirical evidence to the field of positive psychology. The findings highlight that fostering sustainable resilience demands optimized practicum environments, advocating for systemic, strengths-based interventions to protect the human potential of future educators through structurally supported and time-calibrated practicums.

## 1 Introduction

Psychological resilience has emerged as a foundational construct within the field of positive psychology, capturing the dynamic capacity of individuals to adapt, grow, and thrive in the face of adversity. In the context of early childhood education (ECE), the transition from academic training to practical teaching represents a highly demanding phase. Recent positive psychology research emphasizes that teacher resilience is not a fixed trait, but rather a multidimensional process resulting from the continuous interplay between personal cognitive capacities and systemic contextual supports (Song et al., 2026). During the teaching practicum, pre-service teachers encounter real-world pedagogical and emotional challenges that intensely test their psychological resilience (Dekeyser et al., 2025). Rather than focusing exclusively on psychological deficits or occupational burnout, a strength-based positive psychology perspective highlights the importance of reinforcing teachers’ internal strengths and exploring how they utilize available organizational resources to sustain Teachers’ Job Satisfaction and engagement (Yang, 2026).

Despite the critical role of the practicum, a prevalent assumption in higher education persists: the linear belief that longer internships inherently yield better professional outcomes (Cohen et al., 2013). Driven by this “more is better” ambition, many ECE programs have implemented extended practicum periods. However, from a resilience-building perspective, excessively prolonging the physical duration of an internship (Internship Length) without adequate structural mentoring transforms it into a severe systemic risk factor. Without calibrated boundaries, extended practicums impose prolonged systemic stress, demanding sustained emotional and physical effort that is associated with the depletion of pre-service teachers’ psychological resources. Therefore, it is imperative to investigate how this temporal contextual demand interacts with protective environmental resources to relate to the resilience levels of novice teachers.

To unpack these complex interactions, this study adopts the Job Demands-Resources (JD-R) model (Bakker and Demerouti, 2017), reinterpreting it as a Job Demands-Resources (JD-R) model highly aligned with positive psychology principles. Within this framework, job resources are defined as physical, psychological, social, or organizational aspects that not only reduce job demands but also stimulate personal growth and development (Schaufeli and Taris, 2014). Specifically, we identify Person-Organization (P-O) fit—defined as the congruence between an individual’s personal values and the normative culture of their institution—as a crucial protective contextual resource. Recent literature integrating positive psychology with the JD-R theory indicates that organizational fit functions as a fundamental contextual resource that reduces psychological strain and facilitates adaptive coping (Wnuk and Chudzicka-Czupała, 2026). When pre-service teachers experience strong environmental alignment, this resource facilitates meaning in their work and significantly fosters teachers’ job satisfaction (Wnuk and Chudzicka-Czupała, 2026).

Furthermore, consistent with the JD-R theory, we conceptualize Teaching Self-Efficacy (TSE) as a crucial personal resource. Rather than treating resilience as a static trait, we view the mobilization of TSE as part of a resilience-building process that empowers pre-service teachers to confidently navigate the demands of their practicum. Self-efficacy translates individual strengths into long-term occupational joy, and recent empirical studies demonstrate that educators’ psychological capital is positively associated with their teachers’ job satisfaction through the crucial mediating effect of self-efficacy (Yang, 2026). A supportive environment characterized by high P-O fit acts as a psychological catalyst that activates and scaffolds this internal resilience, empowering pre-service teachers to confidently navigate the demands of their practicum.

Despite growing recognition of these dynamics, there is an urgent need to systematically evaluate how systemic risk factors and protective resources collectively shape resilience across critical career transitions. Addressing this gap, the present study utilizes empirical data from 497 pre-service ECE teachers to elucidate these pathways. Specifically, we aim to examine how P-O fit acts as a protective resource that is positively associated with teachers’ job satisfaction, explore the mediating role of TSE as an internal resilience correlate, and critically evaluate the suppressive moderating effect of excessive internship length. By revealing the resource-depleting nature of excessive internship duration, this study contributes valuable empirical evidence to the positive psychology literature, advocating for structurally supported and time-calibrated practicum environments that sustainably protect the human potential of future educators. To guide this empirical investigation through its exploratory and context-sensitive scope, the present study addresses the following three explicit research questions (RQs):

*RQ1*: To what extent is Person-Organization (P-O) fit associated with the job satisfaction of pre-service early childhood education teachers during their teaching practicum?

*RQ2*: How does teaching self-efficacy (TSE) cross-sectionally mediate the relationship between P-O fit and pre-service teachers' job satisfaction?

*RQ3*: What is the functional trajectory of internship length (IL) regarding pre-service teachers' teaching self-efficacy, and does it exhibit a non-linear boundary threshold that challenges the traditional linear 'more is better' assumption within the Chinese institutional context?"

## 2 Literature review and hypotheses development

### 2.1 Psychological resilience and the JD-R framework in pre-service training

The sustainable development of high-quality early childhood education heavily relies on the psychological well-being and professional resilience of its educators (Viac and Fraser, 2020). However, the critical transition from academic training to real-world teaching environments is often fraught with high risks of occupational burnout and attrition (Kim and Asbury, 2020). To fully conceptualize this transition, it is essential to first acknowledge that teacher occupational well-being (TOWB) is not a monolithic state, but a highly multidimensional construct encompassing cognitive, affective, physical, and social dimensions (Hascher and Waber, 2021; Viac and Fraser, 2020). Recent systematic reviews consistently distinguish between the negative dimensions of occupational functioning—such as stress, emotional exhaustion, and burnout—and the positive dimensions, which include work engagement, psychological flourishing, and job satisfaction (Kurrle and Warwas, 2025). Furthermore, contemporary scholarship clarifies the critical distinctions among related psychological constructs: while coping refers to reactive behavioral responses to immediate stressors, resilience represents the proactive, dynamic process of mobilizing personal and contextual resources to maintain professional functioning (Kurrle and Warwas, 2025). Within the Job Demands-Resources (JD-R) framework, these dimensions operate through dual pathways. Chronic job demands trigger a health-impairment process associated with emotional exhaustion, whereas abundant organizational resources activate a motivational process that fosters flourishing (Bakker and Demerouti, 2017; Chan et al., 2024). By specifically examining Teachers’ Job Satisfaction (TJS)—a core positive cognitive-affective dimension of TOWB—this study deliberately focuses on the motivational pathway, empirically evaluating how the resilience-building process translates external structural resources into sustainable professional fulfillment during the practicum (Wang J. et al., 2026). While the practicum is a cornerstone for bridging the theory-practice gap, pre-service teachers navigating this phase frequently encounter practicum-related demands that cause severe stress and emotional exhaustion (Dekeyser et al., 2025).

To unpack the underlying mechanisms of these challenges through a positive psychology lens, this study adopts the Job Demands-Resources (JD-R) model as a robust theoretical framework (Bakker and Demerouti, 2017). According to JD-R theory, job characteristics can be categorized into job demands, which require sustained effort and drain energy, and job resources, which stimulate motivation and buffer psychological costs (Bakker and Demerouti, 2017). Within educational settings, recent scholarship has extensively applied the JD-R framework to understand teacher well-being and emotional exhaustion (Hascher and Waber, 2021). For educators, job demands frequently manifest as intense emotional labor, heavy workloads, and complex classroom management challenges, which are rapidly associated with the depletion of cognitive and emotional reserves. Conversely, crucial teacher-specific job resources include supportive school climates, administrative backing, and effective mentoring (Skaalvik and Skaalvik, 2018). In the context of resilience-building, shifting the focus towards how pre-service teachers harness these organizational job resources is essential for understanding how they protect themselves from early-career burnout and sustain their Teachers’ Job Satisfaction (Song et al., 2026).

### 2.2 Person-organization fit as a protective contextual resource

The concept of Person-Organization (P-O) fit fundamentally describes the compatibility between an individual and their work environment, occurring when an individual’s characteristics are well-matched with the organization’s values, norms, and culture (Kristof-Brown et al., 2005). In the context of early childhood education, the practicum serves as the first immersive organizational socialization process. From the perspective of the JD-R model, a high level of P-O fit acts as a substantial “job resource” (Lesener et al., 2019).

Previous organizational research has consistently demonstrated that when employees perceive a high congruence between their personal values and their organization’s culture, they report significantly higher teachers’ job satisfaction, organizational commitment, and overall job satisfaction (Chen et al., 2016; Westerman and Cyr, 2004). However, in educational settings, P-O fit carries unique pedagogical and developmental significance. It goes beyond mere administrative compliance, reflecting the deep value congruence between a teacher’s personal educational philosophy and the school’s pedagogical culture and normative practices (Zang and Chen, 2022). For pre-service teachers, entering a practicum environment with a highly misaligned pedagogical culture is closely linked to severe ‘reality shock’ and professional identity conflict, significantly exacerbating occupational stress. Specifically within educational settings, empirical studies indicate that pre-service teachers who experience alignment with their internship kindergarten’s pedagogical beliefs experience reduced cognitive dissonance and job stress (Wartini et al., 2023). Viewed through the JD-R lens, this resource facilitates meaning in work and protects them from motivation loss (Wnuk and Chudzicka-Czupała, 2026). According to the motivational process of the JD-R model, such abundant job resources fulfill basic psychological needs. More importantly, from a psychological mechanism perspective, high P-O fit significantly reduces the need for ‘surface acting’ and emotional labor. When pre-service teachers’ pedagogical values align with the institution, they experience less cognitive dissonance and do not need to constantly suppress their authentic teaching beliefs to comply with institutional mandates (Yin et al., 2019). This reduction in emotional exhaustion directly preserves their affective energy, thereby being positively linked to a higher level of teachers’ job satisfaction (TJS).

While P-O fit captures the baseline alignment between individual values and organizational culture, recent scholarship highlights that it operates within a broader ecosystem of contextual resources, particularly school climate and mentoring support (Harrison et al., 2025). Within the practicum setting, a supportive school climate acts as a crucial antecedent to institutional fit, significantly accelerating pre-service teachers’ professional adjustment and overall well-being by fulfilling their basic psychological needs. Furthermore, contemporary extensions of the JD-R framework emphasize the active role of teachers through job crafting, illustrating how educators actively mobilize these contextual resources to navigate job demands and sustain their occupational functioning (Zheng et al., 2024). However, for pre-service teachers, the ability to mobilize such resources is heavily contingent upon mentoring quality. High-quality supervisory support during the practicum provides the critical scaffolding that translates a generalized school climate into personalized, actionable feedback, ultimately fostering their intrinsic motivation and professional identity (Yang and Chen, 2025). By conceptualizing P-O fit alongside these contextual analogues, this study acknowledges that the protective power of organizational alignment is dynamically sustained through a combination of structural school climate, high-quality mentoring, and the active resource mobilization of student teachers.

*Hypothesis 1 (H1)*. Person-organization (P-O) fit is positively and significantly associated with the job satisfaction of pre-service early childhood teachers during their practicum.

### 2.3 The mediating pathway of teaching self-efficacy as internal resilience

Teaching Self-Efficacy (TSE) refers to educators’ beliefs in their own capabilities to organize and execute the courses of action required to successfully accomplish specific teaching tasks (Tschannen-Moran et al., 1998). Within the framework of educator resilience, TSE is widely recognized as a core psychological capital that buffers against workplace stress (Skaalvik and Skaalvik, 2011) and empowers pre-service teachers to confidently navigate the practical demands of the teaching profession (Simaremare et al., 2025).

The Association between P-O fit and TSE: The JD-R model posits that external organizational resources are crucial for cultivating internal psychological resources. Empirical evidence suggests that supportive organizational climates and value congruence provide a psychologically “safe” space for pedagogical experimentation. When operating in an environment with high P-O fit, novice teachers are more likely to receive positive verbal persuasion and experience successful teaching interactions (mastery experiences)—both of which are primary antecedents of self-efficacy identified in social cognitive theory (Zee and Koomen, 2016). Furthermore, organizational alignment validates the novice teacher’s emerging professional identity. When their pedagogical instincts are culturally endorsed by the kindergarten administration, it accelerates their psychological transition from a ‘novice student’ to a ‘capable educator’, directly fostering robust capability beliefs. Recent empirical studies on educators further confirm that organizational support and person-job fit directly and positively relate to teacher self-efficacy (Yang, 2026).

*Hypothesis 2 (H2)*. Person-organization (P-O) fit is positively related to pre-service teachers' teaching self-efficacy (TSE).

The Association between TSE and teachers’ job satisfaction: The link between teaching self-efficacy and job satisfaction is robustly supported by empirical literature (Kariou et al., 2021; Wang J. et al., 2026; Zee and Koomen, 2016). Pre-service teachers equipped with strong self-efficacy are more likely to employ deep emotional regulation strategies and perceive classroom challenges as manageable tasks rather than insurmountable threats. High-efficacy teachers appraise classroom disruptions or heavy workloads as solvable challenges rather than personal failures. This positive cognitive appraisal prevents stress proliferation and effectively reduces emotional exhaustion, allowing them to derive genuine fulfillment from their pedagogical interactions (Zee and Koomen, 2016).

*Hypothesis 3 (H3)*. Teaching self-efficacy (TSE) is positively and significantly associated with pre-service teachers' job satisfaction (TJS).

The Mediation Effect: Recent studies emphasize the substantial power of self-efficacy as a mediating variable in understanding how resilience impacts educator well-being and engagement (Yang, 2026). According to recent empirical applications of the JD-R model, external job resources (like P-O fit) do not solely directly impact well-being; they must be internalized into psychological resilience (such as self-efficacy) to sustainably support higher subjective well-being (Yang, 2026). This conceptualization aligns well with the ‘resource caravan’ principle of the JD-R model, which posits that environmental resources (P-O fit) act as distal predictors that cultivate proximal personal resources (TSE) (Xanthopoulou et al., 2006). We hypothesize that the external environmental advantage provided by P-O fit does not bypass the individual’s cognition; rather, it provides the fertile organizational ground necessary to cultivate the internal confidence (TSE) required to navigate practicum challenges, which is ultimately linked to higher job satisfaction.

While the static relationship between teaching self-efficacy (TSE) and professional outcomes is well-documented, recent longitudinal studies highlight that TSE is not a fixed trait but a dynamic, malleable construct that undergoes significant development during the practicum (Dixon et al., 2026). The developmental trajectory of student teachers’ self-efficacy is heavily contingent upon specific contextual antecedents, such as the provision of structured inquiry opportunities and targeted mentoring feedback, which help them accurately calibrate their competence in the face of increasing task magnitude. Furthermore, the development of TSE is intimately intertwined with emotional adjustment. Longitudinal evidence reveals a critical resource-regulation-belief pathway, wherein the accumulation of psychological resources and adaptive emotion regulation directly predicts subsequent increases in teacher self-efficacy (Zhang and Zhang, 2026). As student teachers learn to regulate the intense emotional demands of field placements, this heightened self-efficacy buffers against emotional exhaustion and burnout, thereby translating early coping processes into sustained occupational satisfaction (Li, 2023). By positioning TSE as a developmental mediator, this study captures how initial contextual support—such as P-O fit—is internalized over time, enabling student teachers to maintain their well-being and job satisfaction throughout the practicum.

*Hypothesis 4 (H4)*. Teaching self-efficacy (TSE) significantly mediates the relationship between P-O fit and teachers' job satisfaction.

### 2.4 Internship length as a systemic risk factor: debunking the “more is better” fallacy

To fully understand the systemic nature of these practicum demands and the persistence of the ‘more is better’ paradigm, it is crucial to contextualize the unique institutional evolution of early childhood teacher education in China. A substantial proportion of higher education institutions currently offering undergraduate early childhood education programs were historically upgraded from vocational junior colleges or normal schools (Li et al., 2016). Historically, these institutions operated under a vocational paradigm that prioritized intensive, long-term practical training to maximize technical proficiency. Although the transition to undergraduate status theoretically mandates a curricular shift toward deeper conceptual grounding, many institutional administrators continue to exhibit historical path dependency (Qi et al., 2024; Zhang et al., 2020). Consequently, they default to or prolong extended practicum requirements, occasionally even promoting these long-duration internships as a distinct institutional pedigree. When this internal administrative inertia intersects with the external reality of preschool labor shortages—where placement kindergartens heavily rely on pre-service teachers as low-cost supplementary staff to manage heavy workloads (Liu and Xin, 2026; Wang J. et al., 2026; Wang X. et al., 2026)—the linear ‘more is better’ assumption becomes structurally reinforced. This unique context explains the substantial variation in practicum duration across programs and underscores why prolonged internships may function more as a systemic demand than a pedagogical resource.

A prominent feature of ECE higher education programs is the implementation of extended practicum periods. The underlying assumption is often a linear “more is better” paradigm: a longer internship inevitably provides more opportunities for practice, thereby enhancing professional development (Cohen et al., 2013). However, without adequate mentor support—which is identified as a crucial resource during practicums (Dekeyser et al., 2025)—we challenge this assumption by conceptualizing excessive Internship Length (IL) as a systemic condition that amplifies chronic job demands. Recent scholarship emphasizes that the practicum is a period of intense vulnerability to emotional exhaustion, particularly when the demands of teaching outpace the available structural supports (Hascher and Waber, 2021). High-quality mentoring is consistently identified as the most critical job resource during this phase, providing novice educators with the necessary emotional scaffolding and pedagogical feedback to navigate practicum shock (Dekeyser et al., 2025; Kim and Asbury, 2020). When extended internships operate in the absence of such robust mentoring, the practicum devolves from a developmental learning opportunity into a source of chronic occupational fatigue. Recent scholarship emphasizes that the teaching practicum is not merely a period of pedagogical skills acquisition, but a highly emotional experience characterized by continuous well-being fluctuations and intensive emotional demands (Chiva-Bartoll et al., 2026; Hartl et al., 2022). During field experiences, pre-service teachers navigate a complex emotional landscape. Recent network analyses reveal a frequent “emotional co-occurrence,” where positive emotions like excitement and interest simultaneously exist alongside high levels of stress, anxiety, and ambivalence (Dreer-Goethe, 2024).

Initially, this emotional complexity and the presence of positive experiences can facilitate psychological adaptation. However, assessing the psychological costs of extended placements reveals a severe shift in these emotional patterns. As the practicum duration extends without adequately calibrated structural support, the emotional demands—such as intense emotional labor and chronic workload—begin to outpace the student teachers’ emotional competencies and coping mechanisms (Dekeyser et al., 2025). Longitudinal tracking indicates that while emotional exhaustion may be buffered initially, it significantly increases as student teachers are forced to confront sustained, unmitigated academic and occupational demands over a prolonged period (Hartl et al., 2022). This specific emotional adaptation trajectory—from initial mixed-emotion resilience to eventual exhaustion—provides a crucial theoretical foundation for interpreting the non-linear boundaries of practicum duration. Furthermore, prolonged exposure to teaching duties without adequate structural mentoring diminishes pre-service teachers’ emotional and cognitive energy (Dekeyser et al., 2025; Ma et al., 2021).

**Figure 1:**
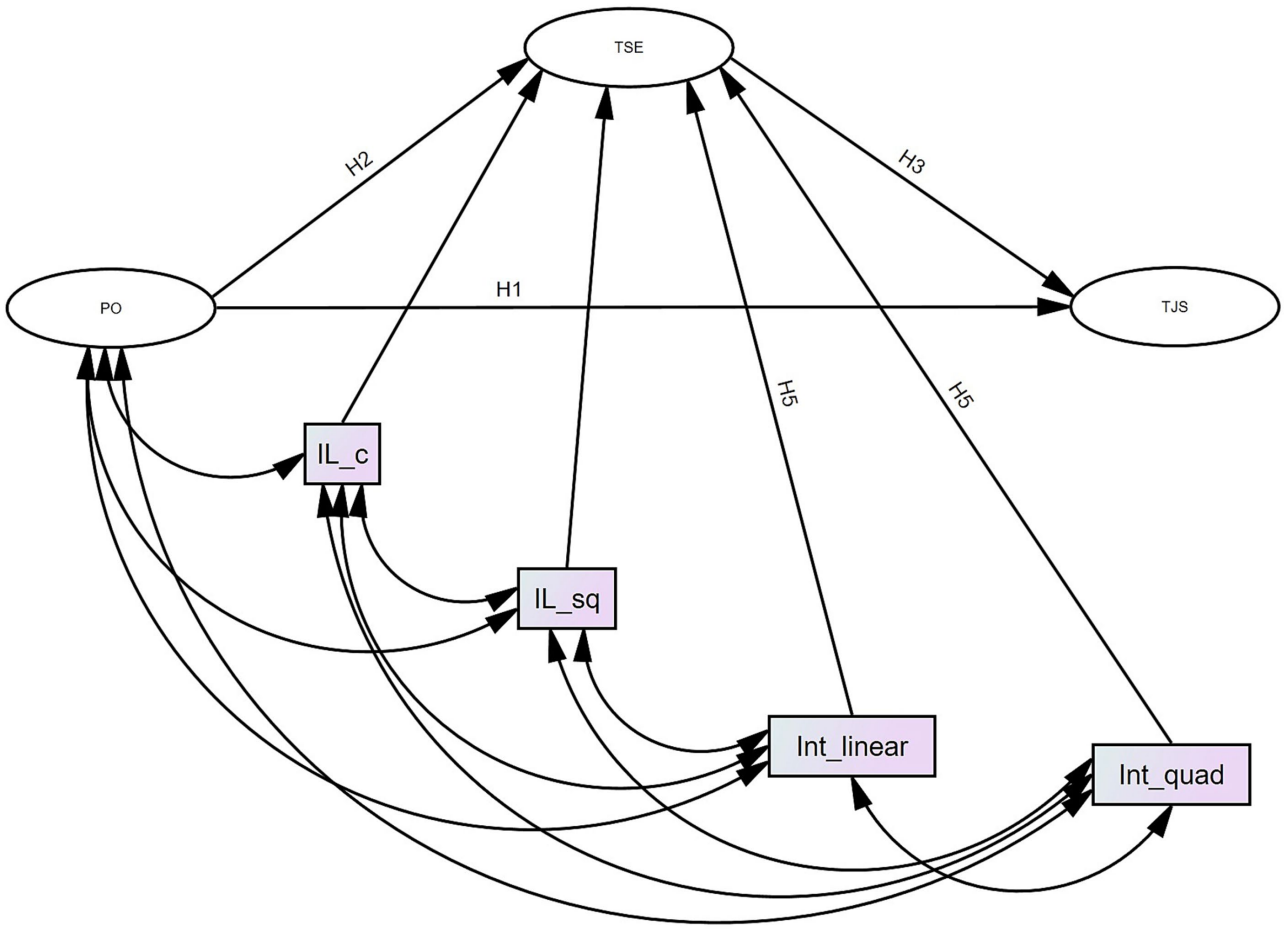
Structural model.

Integrating this empirical evidence into the JD-R model, we hypothesize a complex, non-linear dynamic. While strong organizational alignment (P-O fit) serves as an essential job resource that provides a buffering capacity against initial practicum stress, the prolonged accumulation of daily teaching responsibilities over extended weeks is conceptualized as a chronic systemic demand. This non-linear shift can be understood through the Conservation of Resources (COR) theory (Hobfoll et al., 2018). Initially, the practicum represents a ‘resource-gaining’ phase characterized by rapid pedagogical learning and novelty. However, as the learning curve plateaus, the chronic physical and emotional demands of functioning as supplementary staff begin to outpace the pre-service teachers’ resource replenishment capabilities. We posit that while P-O fit offers linear protective buffering (providing a larger initial resource pool to delay the onset of exhaustion), it cannot infinitely counteract the overwhelming temporal demands of an excessive internship. Ultimately, the protracted strain will govern the overarching trajectory, leading to a non-linear depletion of psychological reserves (Figure 1).

*Hypothesis 5a (H5a)*. There is an inverted U-shaped non-linear relationship between internship length (IL) and teaching self-efficacy; specifically, self-efficacy increases up to an optimal temporal threshold but begins to decline as the duration extends excessively.

*Hypothesis 5b (H5b)*. Person-organization (P-O) fit linearly moderates this relationship. Specifically, P-O fit acts as a temporal buffer that enhances teaching self-efficacy against initial practicum demands, although it cannot alter the overarching inverted U-shaped exhaustion trajectory.

## 3 Materials and methods

### 3.1 Participants and procedure

This cross-sectional study was conducted among pre-service early childhood teachers enrolled in a four-year undergraduate program at a normal university in Hunan Province, China. Data collection took place between April 2024 and June 2024 via an online questionnaire platform. A purposeful sampling method was employed to recruit third- and fourth-year university students (aged 18 to 22 years) who were either currently participating in or had recently completed their educational practicum.

Prior to the survey, all participants were provided with an informed consent form detailing the study’s purpose, the voluntary nature of their participation, and the strict confidentiality of their responses. Participants were informed that they could withdraw from the study at any time without penalty and that no financial compensation would be provided. The research protocol and questionnaire were reviewed and approved by the Ethics Committee of the School of Preschool Education at Changsha Normal University.

Initially, 590 questionnaires were collected. To ensure data quality, a rigorous data cleaning process was implemented (Hair et al., 2012). Respondents who reported an internship length of “0 weeks” were excluded, leaving 524 responses. Subsequently, 21 incomplete questionnaires (accounting for 4.01% of the sample) and 6 questionnaires displaying identical response patterns or extreme outliers (0.01%) were removed. The final valid sample consisted of 497 participants, yielding an effective response rate of 84.2%. Demographically, the final sample included 55 (11.07%) male participants and 442 (88.93%) female participants, which accurately reflects the typical gender distribution in early childhood education programs in China.

### 3.2 Measures

All measurement scales used in this study were originally developed in English and had been previously translated and validated in the Chinese educational context. To ensure contextual appropriateness, minor modifications were made to the wording of some items based on discussions with three domain experts (professors in higher and vocational education), thereby guaranteeing the face and content validity of the instruments. All items were rated on a 7-point Likert scale, ranging from 1 (“strongly disagree”) to 7 (“strongly agree”). For accuracy, a Chinese-English parallel version is provided in the appendix.

Person-Organization (P-O) Fit: P-O fit was assessed using a 5-item scale adapted from existing validated instruments (Cable and DeRue, 2002; Wang and Li, 2023). This scale measures the congruence between the pre-service teachers’ personal values, skills, and the organizational culture and requirements of their internship kindergartens. A sample item is, “My personal values are very similar to the values of the kindergarten where I intern.”

Teaching Self-Efficacy (TSE): The TSE of pre-service teachers was evaluated using a refined 9-item scale. While the original comprehensive scale contained 21 items (Jin, 2020; Liu, 2012), 12 items were excluded during the confirmatory factor analysis (CFA) phase to improve model parsimony and eliminate indicators with low factor loadings or high cross-loadings, thereby achieving optimal convergent validity.

Teachers’ job satisfaction (TJS): TJS was measured using a scale designed to assess the positive emotional and cognitive satisfaction derived from the practicum experience (Lu, 2024; Zhang, 2023). Job satisfaction is widely recognized as a fundamental cognitive and affective dimension of an educator’s broader occupational well-being (Viac and Fraser, 2020). Based on critical reflections regarding construct distinctiveness, initial items reflecting work fatigue and psychological stress were removed to ensure strict construct validity. The refined latent variable comprises precisely targeted items (e.g., ‘I find teaching work very interesting and it’s joyful to be with the children’), demonstrating excellent internal consistency.

Internship Length (IL): Internship length was operationalized as a continuous variable to preserve data variance and allow for rigorous non-linear estimation. Participants were asked to report the exact cumulative number of weeks they had completed in their current kindergarten practicum placement. To test the hypothesized non-linear relationship and curvilinear thresholds, both the mean-centered linear term of internship length (IL_c) and its quadratic squared term (IL_sq) were calculated and utilized in the subsequent polynomial trajectory analysis.

### 3.3 Data analysis

Data analyses were conducted using SPSS 26.0 and AMOS 26.0 software. The analytical procedure followed a systematic structural equation modeling (SEM) approach (Anderson and Gerbing, 1988). First, descriptive statistics and reliability analyses were performed. Following peer-review feedback and initial measurement model assessments, an item refinement process was conducted. Items exhibiting low standardized factor loadings or high residual variances were iteratively removed to optimize the model fit indices and ensure robust convergent and discriminant validity before testing the structural relationships, assessed via Cronbach’s alpha, Composite Reliability (CR), and Average Variance Extracted (AVE) (Hair et al., 2012, 2019). Discriminant validity was verified using the Fornell-Larcker criterion (Fornell and Larcker, 1981).

Second, the structural model was evaluated using maximum likelihood estimation to test the direct hypotheses. Model fit was determined using multiple indices, including the ratio of chi-square to degrees of freedom (χ2/df < 3), Goodness-of-Fit Index (GFI > 0.8), Comparative Fit Index (CFI > 0.9), Root Mean Square Error of Approximation (RMSEA < 0.10), and Standardized Root Mean Square Residual (SRMR < 0.10) (Byrne, 2010; Schreiber et al., 2006).

Third, to test the mediating effect of TSE, a bias-corrected percentile bootstrapping method with 5,000 resamples was applied. A mediating effect was considered statistically significant if the 95% confidence interval (CI) did not include zero (Hayes, 2009; Preacher and Hayes, 2008).

Finally, to rigorously test the non-linear direct effects and boundary conditions of internship duration, a polynomial structural equation modeling (SEM) approach was executed in AMOS 26.0. The structural model incorporated the mean-centered linear term of internship length (IL_c), its quadratic squared term (IL_sq), and their respective linear and quadratic interaction products with P-O fit (Int_linear and Int_quad) to simultaneously evaluate non-linear and conditional moderation mechanisms. The presence of a true inverted U-shaped relationship was statistically evaluated based on the significance of the quadratic path coefficient (IL_sq → TSE), and the precise mathematical inflection point (the vertex) was identified using the standard coordinate formula x = −b/2a following the rigorous methodological guidelines proposed by Lind and Mehlum (2010) and Haans et al. (2016).

Furthermore, because the data were collected through self-reported questionnaires simultaneously, potential Common Method Bias (CMB) was assessed using Harman’s single-factor test. All items from the P-O fit, TSE, and TJS scales were subjected to an unrotated exploratory factor analysis. Finally, demographic characteristics, specifically gender and academic grade, were incorporated as control variables in the structural model to isolate the main effects of the primary constructs.

## 4 Results

### 4.1 Measurement model: reliability and validity

To strictly monitor Common Method Bias (CMB), we employed the Common Latent Factor (CLF) method in AMOS since the Harman’s single-factor test showed a borderline result (51.62%). We compared the standardized regression weights of the model with and without a common latent factor. The differences in regression weights were found to be minimal (all *Δ* < 0.200), indicating that common method bias did not significantly distort the structural relationships in this study. We have also adopted comparison between the CFA single-factor model and the theoretical model. The results show that the fit of the single-factor model (χ^2^/df = 8.514, RMSEA = 0.123, CFI = 0.800, GFI = 0.654, TLI = 0.778) is much worse than that of the theoretical model (χ^2^/df = 1.926, RMSEA = 0.043, CFI = 0.976, GFI = 0.934, TLI = 0.973). Therefore, the results indicate that CMB has no significant effect, the data meets the requirements and can continue to be used. This step ensures the validity of the subsequent CFA and structural models.

Before testing the structural relationships, a Confirmatory Factor Analysis (CFA) was conducted to evaluate the reliability and convergent validity of the measurement model. As shown in Table 1, all constructs demonstrated excellent internal consistency. The Cronbach’s *α* values for Person-Organization fit (PO), Teaching Self-Efficacy (TSE), and teachers’ job satisfaction (TJS) were all above the recommended threshold of 0.80. The Composite Reliability (CR) values ranged from 0.879 to 0.943, exceeding the strict criterion of 0.80, indicating high construct reliability.

**Table 1:** Measurement model: reliability and convergent validity.

Constructs/item	Unstd.	SE	T-Value	P	Std.	SMC	CR	AVE	Cronbach’s alpha
Person–Organization (PO) (Cable and DeRue, 2002; Wang and Li, 2023)							0.879	0.591	0.878
My personal values are very similar to those of the kindergarten where I intern.	1				0.738	0.545			
My values and characteristics can be reflected in a kindergarten.	0.958	0.057	16.669	< 0.001	0.784	0.615			
The work in kindergarten provided me with material and spiritual resources that are highly consistent with what I am looking for.	0.991	0.059	16.661	< 0.001	0.784	0.615			
My personal skills can well meet the needs of kindergarten work.	0.982	0.06	16.478	< 0.001	0.775	0.601			
The education and training I have received are in line with the requirements of kindergarten work.	0.998	0.062	16.214	< 0.001	0.763	0.582			
Teaching Self-Efficacy (TSE) (Jin, 2020; Liu, 2012)							0.943	0.647	0.942
I can apply various teaching methods to design engaging and interesting activities.	1				0.773	0.598			
When designing activities, I can grasp the key points and difficulties of the activity stages.	1.072	0.05	21.507	< 0.001	0.87	0.757			
During the activity, I can effectively extract information from young children’s feedback and stimulate their in-depth thinking.	1.025	0.05	20.401	< 0.001	0.834	0.696			
I can quickly calm down chaotic or noisy situations.	0.999	0.05	19.983	< 0.001	0.821	0.674			
I can stimulate and motivate young children’s enthusiasm for participating in activities.	1.01	0.052	19.326	< 0.001	0.799	0.638			
If some young children are not paying attention, I can help them focus.	0.974	0.051	18.984	< 0.001	0.788	0.621			
During the activity, I can provide young children with ample opportunities to express themselves and make decisions.	1.005	0.052	19.395	< 0.001	0.801	0.642			
I can help children with weaker developmental abilities make progress.	0.943	0.052	18.183	< 0.001	0.761	0.579			
I can provide timely evaluations of young children’s classroom performance to promote their development.	1.005	0.053	18.86	< 0.001	0.784	0.615			
Teachers’ Job Satisfaction (TJS) (Lu, 2024; Zhang, 2023)							0.910	0.591	.0.909
I am satisfied with the teaching conditions in the kindergarten.	1				0.76	0.578			
I am satisfied with the interpersonal relationships among colleagues.	1.074	0.059	18.138	< 0.001	0.792	0.627			
I feel happy to see the growth of the children.	0.84	0.054	15.696	< 0.001	0.696	0.484			
My efforts can receive recognition from others.	1.04	0.059	17.568	< 0.001	0.77	0.593			
I find teaching work very interesting and it’s joyful to be with the children.	1.042	0.058	18.024	< 0.001	0.787	0.619			
My interactions with the children are positive and harmonious, bringing me a sense of physical and mental well-being.	1.049	0.055	19.144	< 0.001	0.83	0.689			
I enjoy the process as the children respond positively to my efforts.	0.989	0.059	16.744	< 0.001	0.738	0.545			

SE, Standard Error; P, *p*-value; SMC, Squared Multiple Correlations; CR, Composition reliability; AVE Average variance extracted. ***p < 0.001. PO, Person-Organization Fit; TSE, Teaching Self-Efficacy; TJS, Teachers’ Job Satisfaction; [R] = indicates reverse score items.

Additionally, a standalone one-factor Confirmatory Factor Analysis (CFA) for the Person-Organization (P-O) fit scale demonstrated strong empirical fit, successfully validating the consolidation of its items into a single, holistic contextual resource variable for the structural model.

Convergent validity was confirmed as the Average Variance Extracted (AVE) values for all constructs ranged from 0.591 to 0.647, surpassing the acceptable level of 0.50. Furthermore, all standardized factor loadings (Std.) were significant (*p* < 0.001) and greater than the 0.60 threshold, and the Squared Multiple Correlation (SMC) values were all above 0.36.

Discriminant validity was assessed using the Fornell-Larcker criterion. As presented in Table 2, the square root of the AVE for each construct (bolded on the diagonal) was greater than its correlation coefficients with other latent variables in the model, establishing satisfactory discriminant validity.

**Table 2:** Discriminant validity (Fornell and Larcker, 1981).

Constructs	Mean	SD	TSE	TJS	PO
TSE	4.611	1.185	**0.804**		
TJS	4.543	1.204	0.760	**0.769**	
PO	4.032	1.511	0.617	0.688	**0.769**

Bold values on the diagonal represent the square root of the AVE. Off-diagonal values are the correlations between constructs.

### 4.2 Structural model and hypothesis testing

The overall fit of the structural equation model (SEM) was evaluated. The results indicated that the proposed model fit the empirical data well: *χ2*/df = 1.926 (which is < 3), GFI = 0.934, RMSEA = 0.043, CFI = 0.976, NFI = 0.951, AGFI = 0.917, and SRMR = 0.033. The fit indices met the recommended criteria, indicating an acceptable model fit (Table 3).

**Table 3:** Model fit indices.

Fit indices	χ2	DF	*χ2/DF*	GFI	RMSEA	CFI	NFI	AGFI	SRMR
Reference value	-	-	<3	>0.9	<0.08	>0.9	>0.9	>0.9	<0.08
Mediation Model	358.212	186.000	1.926	0.934	0.043	0.976	0.951	0.917	0.033
Polynomial Non-linear Model	448.701	258	1.739	0.931	0.039	0.979	0.953	0.913	0.032

χ^2^ Chi-Square Minimum Fit Function, DF Degrees of Freedom, *χ^2^/DF* Chi-Square to Degrees of Freedom ratio, GFI Goodness of Fit Index, AGFI Adjusted Goodness of Fit Index, CFI Comparative Fit Index, RMSEA Root Mean Square Error of Approximation, NFI Normed Fit Index, SRMR Standardized Root Mean Square Residual.

The structural path analysis revealed the main effects among the variables (Table 4). The results supported Hypothesis 1, showing that P-O fit had a significant positive direct effect on TJS (*β* = 0.353, *p* < 0.001). Furthermore, TSE significantly and positively predicted TJS (*β* = 0.543, *p* < 0.001), supporting Hypothesis 2. The path from P-O fit to TSE was also significant and positive (*β* = 0.617, *p* < 0.001), lending support to Hypothesis 3.

**Table 4:** Hypothesis testing results (main effects).

Hypotheses	Constructs	Unstandardized path coefficient	S.E.	C.R.	*P*	Standardized path coefficient (β)	Decision
Model with mediators							
H1	PO → TJS	0.285	0.039	7.303	< 0.001	0.353	Supported
H2	TSE → TJS	0.535	0.050	10.618	< 0.001	0.543	Supported
H3	PO → TSE	0.503	0.044	11.469	< 0.001	0.617	Supported

*** *P* < 0.001.

These structural relationships remained significant after controlling for the participants’ gender and academic grade level. The analysis indicated that the control variables did not significantly confound the main pathways, confirming the robustness of the hypothesized model.

### 4.3 Mediation analysis

To test the mediating effect of Teaching Self-Efficacy (TSE) proposed in Hypothesis 4, a bias-corrected percentile bootstrapping method with 5,000 resamples was performed. As detailed in Table 5, the standardized indirect effect of PO on TJS via TSE was significant (Indirect Effect = 0.269, *p* < 0.001). The 95% confidence intervals (Bias-Corrected CI [0.206, 0.354]; Percentile CI [0.203, 0.350]) did not include zero, confirming the statistical significance of the mediation. The indirect effect accounted for 48.56% of the total effect (Total Effect = 0.554), indicating a robust partial mediation mechanism. Thus, Hypothesis 4 was supported.

**Table 5:** Bootstrapping results for the mediation effect.

SIE	Point estimate	Product of coefficients		Bootstrapping = 5,000				Ratio %
				Bias-corrected 95% CI		Percentile 95% CI		
		SE	Z	Lower	Upper	Lower	Upper	
Indirect effect								
PO-TSE-TJS	0.269	0.037	7.270	0.206	0.354	0.203	0.350	48.56%
Direct effect								
PO-TJS	0.285	0.042	6.79	0.209	0.374	.0.206	0.371	51.44%
Total effect								
PO-TJS	0.554	0.056	9.89	0.454	0.678	0.449	0.671	100.00%

### 4.4 Non-linear effects and polynomial trajectory analysis

In contrast to previous studies that often categorise practicum duration into arbitrary distinct phases, internship length was operationalised as a continuous variable in this study. Participants reported the exact number of weeks they had completed in their current kindergarten placement at the time of the survey (Table 6). The reported durations ranged continuously from 2 to 19 weeks (Mean = 12.21, SD = 4.397). Treating internship length as a continuous variable preserves the variance of the raw data, averts the statistical power loss associated with artificial dichotomisation, and importantly, allows for the precise mathematical estimation of non-linear curvilinear thresholds.

**Table 6:** Descriptive statistics of internship length (IL).

Internship length	Frequency (*n*)	Percentage (%)
2 weeks	2	0.604
3 weeks	4	0.805
4 weeks	3	0.604
5 weeks	22	4.427
6 weeks	37	7.445
7 weeks	31	6.237
8 weeks	27	5.433
9 weeks	28	5.634
10 weeks	35	7.042
11 weeks	21	4.225
12 weeks	16	3.219
13 weeks	42	8.451
14 weeks	43	8.652
15 weeks	38	7.646
16 weeks	61	12.274
17 weeks	23	4.628
18 weeks	33	6.64
19 weeks	30	6.036

To rigorously test the non-linear association of internship length (IL) on pre-service teachers’ teaching self-efficacy (TSE) and to empirically challenge the linear “more is better” paradigm, a polynomial structural equation modelling (SEM) approach was executed. The model incorporated the mean-centred linear term of internship length (IL_c), its quadratic squared term (IL_sq), and the two corresponding product terms (Int_linear and Int_quad) to simultaneously evaluate potential non-linear moderation effects. The overall structural model demonstrated a satisfactory fit (Table 3) to the empirical data (χ^2^/df = 1.739, CFI = 0.979, RMSEA = 0.039), providing a robust baseline for path coefficient evaluation.

**Figure 2:**
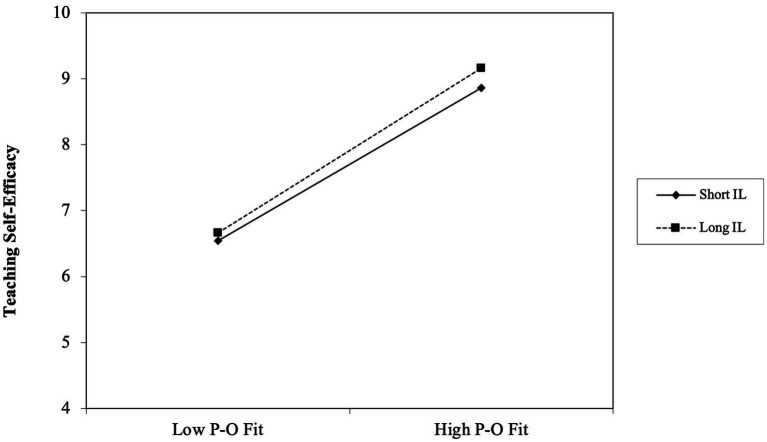
Simple slope plot.

The statistical estimation (Table 7) revealed that while the linear interaction effect (Int_linear → TSE: β = 0.060, S.E. = 0.013, C.R. = 4.566, *p* < 0.001) was positively significant. Simple slope analysis indicated that the positive association between P-O fit and teaching self-efficacy was enhanced under longer internship durations, suggesting that high value congruence functions as a crucial temporal buffer to cushion student teachers during initial field exposure (Figure 2). However, the quadratic interaction effect (Int_quad → TSE: β = −0.004, S.E. = 0.002, C.R. = − 1.591, p = 0.112) did not reach statistical significance. These results indicate that although person-organization fit (PO) linearly buffers the temporal demands of internship duration on teaching self-efficacy, it cannot alter the fundamental curvature of the overarching trajectory.

**Table 7:** Path coefficients of the non-linear structural model.

Path links	Unstandardized estimate	S.E.	C.R.	*p*-value	Statistical decision
PO → TJS	0.527	0.120	4.412	< 0.001	Supported
TSE → TJS	0.506	0.055	9.142	< 0.001	Supported
PO → TSE	0.766	0.131	5.835	< 0.001	Supported
IL_c → TSE	−0.033	0.016	−2.093	0.036	Supported (Non-linear)
IL_sq → TSE	−0.014	0.004	−3.257	0.001	Supported (Inverted U-shaped)
Int_linear → TSE	0.060	0.013	4.566	< 0.001	Supported
Int_quad → TSE	−0.004	0.002	−1.591	0.112	Not supported

*** *p* < 0.001. PO, Person-Organization Fit; TSE, Teaching Self-Efficacy; TJS, Teachers’ Job Satisfaction; IL_c, Mean-centred linear term of internship length; IL_sq, Quadratic squared term of internship length; int_linear, Linear interaction term; int_quad, Quadratic interaction term.

However, a finding emerged regarding the direct curvilinear trajectory of internship duration. The structural path coefficients indicated that the mean-centred linear term of internship length significantly and negatively associated with teaching self-efficacy (β = −0.033, S.E. = 0.016, C.R. = −2.093, *p* = 0.036). Crucially, the quadratic term of internship length (IL_sq → TSE) exerted a significant negative effect on teaching self-efficacy (β = −0.014, S.E. = 0.004, C.R. = −3.257, *p* = 0.001). The highly significant and negative quadratic coefficient (a = −0.014) confirms the existence of an inverted U-shaped non-linear relationship between practicum duration and teaching self-efficacy, validating that the marginal returns of internship extension flip from asset to liability.

Following the rigorous methodological guidelines proposed by Haans et al. (2016) and Lind and Mehlum (2010) for theorizing and testing inverted U-shaped relationships, we proceeded to locate the exact inflection point (the vertex) where the marginal return of internship length flips from positive to negative. To avoid the pitfall of assuming an inverted U-shape based solely on a significant quadratic term, it is imperative to verify that the theoretical turning point falls well within the actual data range (Lind and Mehlum, 2010). The mean-centred vertex coordinate was calculated using the standard mathematical estimation:


$$x=\frac{-b}{2a}$$

**Figure 3:**
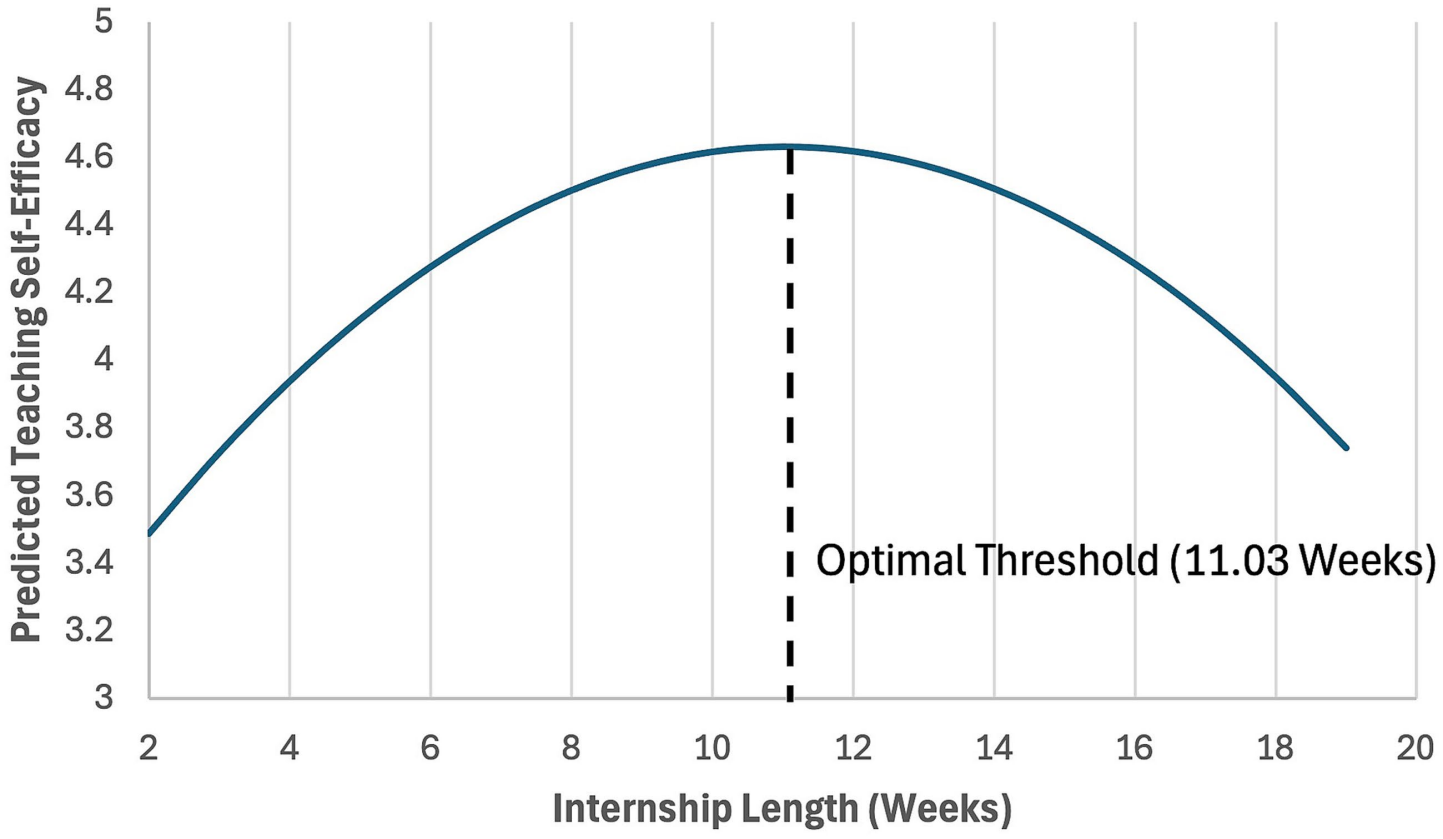
The inverted U-shaped relationship between internship length and teaching self-efficacy.

Where b represents the unstandardized path coefficient of the linear term (IL_c = −0.033) and a represents the coefficient of the quadratic term (IL_sq = −0.014). This calculation yielded a mean-centred coordinate of −1.179. By restoring this value to the original temporal metric based on the sample mean of 12.21 weeks, the definitive optimal threshold was empirically established at 11.03 weeks (12.21 + [−1.179]) (Figure 3). This calculated vertex falls squarely within the empirical data range of our study (2 to 19 weeks), thereby fully satisfying the necessary and sufficient conditions for a true inverted U-shaped relationship (Haans et al., 2016).

## 5 Discussion

Grounded in the Job Demands-Resources (JD-R) model (Bakker and Demerouti, 2017), this study aimed to unpack the complex, context-sensitive mechanisms influencing the psychological resilience and job satisfaction of pre-service early childhood teachers during their critical career transition. Utilizing structural equation modeling and polynomial trajectory analysis on empirical data from 497 pre-service educators, our findings provided a robust, theoretically integrated mapping of these pathways. Specifically, the empirical evidence demonstrated that Person-Organization (P-O) fit functions as a fundamental distal contextual resource that is significantly associated with job satisfaction, both directly and through the mediating pathway of teaching self-efficacy (TSE). Crucially, the investigation challenges the linear “more is better” paradigm of teacher practicum duration. While P-O fit provides a significant linear buffering capacity to cushion temporal demands, the overriding direct trajectory of internship length follows a prominent inverted U-shaped curvilinear path, estimating an empirical turning point at 11.03 weeks. Beyond this critical boundary, excessive temporal demands are associated with non-linear resource depletion and diminished pre-service teachers’ self-efficacy regardless of environmental alignment.

### 5.1 Theoretical implications for psychological resilience and well-being

First, our finding that P-O fit is directly, positively, and significantly associated with teachers’ job satisfaction (*β* = 0.353, C. R. = 7.303, *p* < 0.001) strongly supports Hypothesis 1. This results extends recent ECE organizational scholarship by elucidating the underlying emotional mechanism. In educational settings, value congruence goes beyond administrative compliance; it reflects alignment in pedagogical philosophy. From the lens of emotional labor theory, when pre-service teachers find their pedagogical instincts culturally endorsed by their placement kindergarten, the psychological need for “surface acting” and emotional suppression is significantly minimized. This alignment mitigates cognitive dissonance and preserves affective energy, translating organizational fit directly into occupational well-being and job satisfaction. Consistent with the broader conceptualization of teacher well-being as a multidimensional construct (Kurrle and Warwas, 2025), this finding demonstrates that value alignment is a potent proximal resource. However, as contemporary extensions of the JD-R framework suggest, this alignment does not operate in a vacuum; it is dynamically sustained by the surrounding school climate and mentoring quality (Harrison et al., 2025; Yang and Chen, 2025). When kindergarten environments offer psychological safety, they facilitate the baseline P-O fit to manifest as sustainable cognitive-affective job satisfaction (TJS).

Concurrently, the significant positive associations between P-O fit and TSE (*β* = 0.617, C.R. = 11.469, *p* < 0.001) and between TSE and TJS (*β* = 0.543, C.R. = 10.618, *p* < 0.001) provide empirical validation for Hypothesis 2 and Hypothesis 3, respectively. These pathways resonate with Skaalvik and Skaalvik, illustrating how a supportive organizational climate fosters mastery experiences—the primary antecedent of self-efficacy. More importantly, the psychological bridge from TSE to job satisfaction is deeply rooted in cognitive appraisal theory. High-efficacy pre-service teachers actively appraise classroom disruptions and heavy workloads as solvable pedagogical challenges rather than insurmountable professional threats. This resilient cognitive appraisal blocks stress proliferation, allowing novice educators to derive genuine fulfillment and professional joy from their classroom interactions.

Second, the confirmation of the mediation model supports Hypothesis 4, revealing that the standardized indirect effect of P-O fit on job satisfaction via TSE is highly significant (Indirect Effect = 0.269, *p* < 0.001), accounting for 48.56% of the total effect. This empirical mechanism contributes substantially to positive psychology by mapping the precise transition from external environment to internal cognitive resilience. Rather than bypass individual cognition, distal environmental resources must be psychologically internalized to sustainably generate well-being.

This finding provides a compelling empirical application of the “resource caravan” principle within JD-R theory (Xanthopoulou et al., 2006). In this context, P-O fit acts as a distal, structural environmental catalyst that does not merely sit parallel to well-being, but actively scaffolds and fosters proximal personal resources (TSE). The external advantage of value congruence builds the internal psychological reservoir of confidence required to safely execute pedagogical tasks, ensuring that the mobilization of personal strengths results in a powerful “caravan” of positive occupational outcomes. By confirming the mediating pathway of TSE, our study moves beyond static models to capture a dynamic resource-regulation-belief pathway (Zhang and Zhang, 2026). This supports the developmental view of self-efficacy (Dixon et al., 2026), illustrating that an initial environmental advantage (P-O fit) is gradually internalized over time. Student teachers translate external organizational support into internal professional confidence, which eventually empowers them to regulate emotional demands and achieve long-term professional fulfillment.

Finally, this study offers a critical dialogue with educational policy by deconstructing the traditional “more is better” fallacy through polynomial trajectory analysis, successfully confirming Hypothesis 5a and Hypothesis 5b. The results revealed a highly nuanced temporal dynamic: while the linear interaction term is significantly positive (Int_linear → TSE: *β* = 0.060, C.R. = 4.566, *p* < 0.001), the quadratic main effect of internship length is significantly negative (IL_sq → TSE: *β* = −0.014, C.R. = − 3.257, *p* = 0.001). This empirical confirmation of a curvilinear boundary directly challenges long-standing pedagogical assumptions embedded in teacher education policies (e.g., Cohen et al., 2013), which historically operated under the linear belief that uncalibrated increases in field duration linearly translate into superior professional adaptation and capability. This curvilinear trajectory explicitly echoes recent longitudinal observations regarding pre-service teachers’ well-being fluctuations and the psychological costs of extended placements (Chiva-Bartoll et al., 2026; Hartl et al., 2022). Our empirical threshold (11.03 weeks) provides a precise quantification of when the accumulation of practicum-related emotional demands begins to outpace student teachers’ emotional competencies, driving the transition from initial mixed-emotion resilience to eventual exhaustion. Our non-linear baseline strongly aligns with and provides empirical quantification for the systemic critiques raised by Lawson et al. (2015), who argued that the structural arrangement of the teaching practicum often harbors diminishing returns if physical duration outpaces structural scaffolding.

This interaction can be interpreted using the Conservation of Resources (COR) theory (Hobfoll et al., 2018) and the longitudinal dynamics of the JD-R framework (Lesener et al., 2019). As visualized in the simple slope analysis (Figure 2), during the initial phases of field exposure, high P-O fit functions as a vital structural buffer. Pre-service teachers with strong organizational alignment possess a larger initial resource pool, allowing them to effectively neutralize early practicum shock and adaptively navigate initial environmental demands. However, the non-significant quadratic interaction (*β* = −0.004, *p* = 0.112) coupled with the prominent negative quadratic main effect confirms that P-O fit cannot alter the fundamental curvature of the trajectory; it merely shifts the baseline level of efficacy. This empirical phenomenon demonstrates that while environmental resources can temporarily cushion temporal strain, they cannot permanently stave off the structural deficits of an excessively prolonged internship.

Mathematically locating the estimated threshold at 11.03 weeks exposes the changing resource dynamics across the career transition. According to COR theory, the first 11 weeks function as a “resource-gaining” phase dominated by rapid pedagogical learning, environmental novelty, and organizational socialization. This early phase aligns with the adaptive mastery experiences highlighted by Wang J. et al. (2026) and Wang X. et al. (2026). However, as the learning curve plateaus, the chronic, uncalibrated physical and emotional demands of extended weeks outpace the student teachers’ resource replenishment capabilities, shifting the practicum into a severe “resource loss phase.”

In the specific context of China’s early childhood education system—characterized by institutional path dependency where upgraded undergraduate universities default to extended internships (Qi et al., 2024), intersecting with systemic preschool labor shortages—extended practicums often structurally transform student teachers into low-cost, supplementary labor. When pre-service educators face long hours of repetitive, high-intensity emotional labor without proportional resource replenishment, a maladaptive “resource loss spiral” is initiated. As documented by Zhang et al. (2020), early childhood educators in China navigate highly demanding emotional regulation climates characterized by deep power distances and high collective expectations. When extended past the 11.03-week threshold, these intense occupational demands trigger severe emotional exhaustion and professional identity crises, a phenomenon that strongly resonates with the recent empirical findings of Liu and Xin (2026). Without adequate structural support or mentor-guided resource reciprocity (Dekeyser et al., 2025), this protracted strain rapidly depletes pre-service teachers’ cognitive and psychological reserves, which is linked to a sharp decline in their teaching self-efficacy. Consequently, the temporal factor flips the internship from a developmental opportunity into an exhausting systemic risk (Ma et al., 2021), actively suppressing the positive efficacy-building benefits of organizational fit and underscoring the urgent need for time-calibrated policy designs.

### 5.2 Practical implications: strengths-based systemic interventions

The findings offer concrete, actionable insights for higher education institutions and ECE policymakers aimed at designing strengths-based practicum interventions that protect human potential.

#### 5.2.1 Time-calibrated environments

Institutions must establish an evidence-based “optimal threshold” for internship duration. Echoing the concerns raised by practicum shock literature (Dekeyser et al., 2025), our empirical results indicate a significant suppressive trend: as the practicum duration extends, the positive resource-building effects of organizational fit become increasingly weakened. Particularly when the internship extends into the highest measured category—the “super-long” phase exceeding 11.03 weeks—this systemic demand places the heaviest burden on pre-service teachers. Rather than uncritically pursuing excessively long internships, we advocate calibrating practicum length to a moderate span. This duration appears sufficient for organizational socialization without triggering the severe resource depletion observed in prolonged exposure.

#### 5.2.2 Structurally supported systems

Universities and internship kindergartens must proactively construct targeted structural support systems to buffer the inevitable systemic demands of the practicum. To prevent resource depletion, we suggest three specific operational mechanisms:

- Structured mentoring: Transitioning from passive observation to active co-teaching models, where experienced mentors provide dedicated guidance to help interns navigate emotional and pedagogical challenges (Wartini et al., 2023).
- Formative feedback mechanisms: Replacing infrequent summative evaluations with regular constructive feedback loops, enabling pre-service teachers to promptly consolidate their self-efficacy.
- University-kindergarten collaboration: University supervisors should conduct regular emotional check-ins to monitor signs of occupational fatigue and provide timely psychological scaffolding.

### 5.3 Limitations and future directions

Despite its theoretical and practical contributions, this study has several limitations that warrant acknowledgment and offer directions for future research.

First, the cross-sectional design restricts the ability to establish absolute causal relationships among P-O fit, TSE, and TJS. While our structural model is grounded in robust JD-R theoretical frameworks, the variables were measured simultaneously. Future research should employ longitudinal, cross-lagged, or experimental designs to capture the dynamic fluctuations of pre-service teachers’ well-being across different stages of their practicum and firmly establish causality.

Second, our control variable strategy was limited to demographic factors. We omitted several theoretically relevant institutional variables, such as mentoring quality, daily workload, and specific placement school characteristics. As mentoring support serves as a central protective factor, its absence introduces potential omitted variable bias. Additionally, the study sample exhibits a heavy gender skew (88.93% female). While this authentically aligns with the global demographic reality of the early childhood education workforce, its potential implications for the generalizability of the findings to male pre-service teachers should be noted with caution.

Third, the sample was exclusively drawn from a specific normal university in Hunan Province, China. While this provides valuable localized insights, it limits the generalizability of the findings. The practicum experiences of these pre-service teachers are embedded within the specific context of the Chinese higher education system, which may be characterized by unique mandated practicum lengths and culturally distinct mentor-mentee dynamics (e.g., high power distance and collective educational values). Subsequent studies should aim to replicate this model across broader geographical regions and diverse western or other cultural educational systems to validate the cross-cultural consistency of the “more is better” fallacy.

Finally, although Harman’s single-factor test indicated that common method bias was not a severe issue, the reliance on self-reported questionnaires may still introduce subjectivity. Incorporating multi-informant data (e.g., evaluations from kindergarten mentors or university supervisors) would yield a more objective and comprehensive assessment of pre-service teachers’ developmental outcomes.

## 6 Conclusion

Situated within the global challenge of early-career educator attrition, this study utilizes an integrated JD-R and Conservation of Resources (COR) framework to provide a nuanced understanding of pre-service early childhood teachers’ psychological resilience and adaptation. It highlights Person-Organization (P-O) fit as a critical protective contextual resource that mitigates emotional labor and cultivates teaching self-efficacy as a core cognitive pathway to teachers’ job satisfaction.

Notably, the polynomial trajectory analysis empirically deconstructs the traditional “more is better” fallacy regarding practicum duration. While organizational alignment offers an initial linear buffer against temporal demands, practicums extending beyond the empirically established 11.03-week threshold are highly likely to precipitate a non-linear resource loss spiral, associated with severely depleted pre-service teachers’ self-efficacy.

To cultivate a resilient future ECE workforce, higher educational programs should re-evaluate traditional duration-based practicum designs. Policymakers and institutions must prioritize the quality of organizational fit and structural mentoring over mere physical duration, ensuring that the transition into the teaching profession is a time-calibrated process of resilience-building rather than chronic resource depletion.

## Data Availability

The raw data supporting the conclusions of this article will be made available by the authors, without undue reservation.
