# Supplementary material for: The “more is better” fallacy in pre-service teacher training: a positive psychology perspective on the resilience-building process and job satisfaction
Source: Front Psychol. 2026 Jun 19;17:1885723. doi: 10.3389/fpsyg.2026.1885723 (PMC13328087; doi:10.3389/fpsyg.2026.1885723)
Supplement: Supplementary file 1 [file Table_1.DOCX]

**S1 Table.** Scale English- Chinese Correspondence

| **English** | **Chinese** |
| --- | --- |
| *Person–Organization (PO)* (Cable & DeRue, 2002; Wang Y. & Li, 2023) |  |
| My personal values are very similar to those of the kindergarten where I intern. | 我个人的价值观和我所在的幼儿园的价值观非常相似。 |
| My values and characteristics can be reflected in a kindergarten. | 我的价值观和特质能在幼儿园中得以体现。 |
| The work in kindergarten provided me with material and spiritual resources that are highly consistent with what I am looking for. | 幼儿园工作提供给我的物质和精神资源，和我想找的工作十分契合。 |
| My personal skills can well meet the needs of kindergarten work. | 我个人的技能能够很好地满足幼儿园工作需要。 |
| The education and training I have received are in line with the requirements of kindergarten work. | 我接受的教育及培训与幼儿园工作需要相匹配。 |
| *Teaching Self-Efficacy (TSE)* (Jin, 2020; Liu, 2012) |  |
| I can apply various teaching methods to design engaging and interesting activities. | 我能运用多种教学方法，将活动设计得生动有趣。 |
| When designing activities, I can grasp the key points and difficulties of the activity stages. | 活动设计时，我能把握好活动环节的重难点。 |
| During the activity, I can effectively extract information from young children's feedback and stimulate their in-depth thinking. | 活动中，我能对幼儿反馈的信息进行有效提炼，引发幼儿进行深入的思考。 |
| I can quickly calm down chaotic or noisy situations. | 我能迅速平息活动中的混乱或吵闹情形。 |
| I can stimulate and motivate young children's enthusiasm for participating in activities. | 我能激发、调动幼儿参与活动的积极性。 |
| If some young children are not paying attention, I can help them focus. | 如果某些幼儿不注意听讲，我能使其集中注意力。 |
| During the activity, I can provide young children with ample opportunities to express themselves and make decisions. | 活动中，我能给予幼儿充分表达和做决定的机会。 |
| I can help children with weaker developmental abilities make progress. | 我能让能力发展比较弱的幼儿获得进步。 |
| I can provide timely evaluations of young children's classroom performance to promote their development. | 对于幼儿的课堂表现，我能及时给予评价，促进幼儿发展。 |
| *Teachers' Job Satisfaction (TJS)* (Lu, 2024; Zhang, 2023) |  |
| I am satisfied with the teaching conditions in the kindergarten. | 我对幼儿园的教学条件感到满意。 |
| I am satisfied with the interpersonal relationships among colleagues. | 我对同事间的人际关系感到满意。 |
| I feel happy to see the growth of the children. | 看到孩子们的成长我感到很高兴。 |
| My efforts can receive recognition from others. | 我的付出能得到他人的肯定。 |
| I find teaching work very interesting and it's joyful to be with the children. | 我觉得教学工作很有趣，和孩子们在一起很开心。 |
| My interactions with the children are positive and harmonious, bringing me a sense of physical and mental well-being. | 我与孩子们的互动积极、和谐，使我感到身心愉悦。 |
| I enjoy the process as the children respond positively to my efforts. | 我的付出能够得到孩子们的积极回应，我很享受这样的过程。 |

Note: PO = Person-Organization Fit; TSE = Teaching Self-Efficacy; TJS = Teachers’ Job Satisfaction.
